# Life-threatening pulmonary coinfection with *Mycobacterium tuberculosis* and *Aspergillus lentulus* in a diabetic patient diagnosed by metagenome next-generation sequencing

**DOI:** 10.1186/s12879-023-08052-y

**Published:** 2023-02-09

**Authors:** Hua Zhang, Guangming Liu, Lin He, Yun Zhu, Haobo Tu, Shifang Zhuang

**Affiliations:** 1 Department of Intensive Care Medicine, Pidu District People’s Hospital, Chengdu, China; 2 Genskey Medical Technology Co., Ltd, Beijing, China

**Keywords:** *Mycobacterium tuberculosis*, *Aspergillus lentulus*, Diabetes mellitus, Pneumonia, Coinfection, mNGS

## Abstract

**Background:**

China has a double burden of diabetes mellitus and tuberculosis. Diabetes mellitus and tuberculosis are both important risk factors for *Aspergillus* infection. *Aspergillus lentulus* is an emerging fungal pathogen in China and invasive aspergillosis due to *A. lentulus* is associated with high mortality.

**Case presentation:**

A 79-year-old man was admitted to our hospital, complaining of a 7-day history of fever. Five days before admission, he was diagnosed with pulmonary infection at a local hospital, but his symptoms did not relieve after antibiotic therapy. The patient was diagnosed with diabetes mellitus two months ago. About 20 days ago, he began to present chest tightness and shortness of breath after physical activity. After admission, he developed continuous fever and rapid respiratory deterioration, and finally died after his family abandoned treatment. Pulmonary coinfection with *M. tuberculosis* and *A. lentulus* was identified by metagenome next-generation sequencing (mNGS) from bronchoalveolar lavage fluid.

**Conclusions:**

Clinicians and laboratories should be alert to the emerging *A. lentulus* infection in China due to its drug-resistance and high mortality. In comparison with conventional methods, mNGS has a great advantage for the diagnosis of mixed pulmonary infection.

## Background

There is a double burden of diabetes mellitus (DM) and tuberculosis (TB) in China: the TB cases account for 7.4% of the global total in 2021; the prevalence of DM has increased from less than 1% in 1980 to 11.2% in 2017 [1, 2]. Strong evidence has proved the association between the two diseases. In patients with active TB, DM can lead to a poor TB treatment outcome, a higher chance of relapse, and an increased risk of death [3].

TB and DM are both important risk factors for *Aspergillus* infection. Coinfection with *M. tuberculosis* and *Aspergillus* is common in Asia and Africa countries. The combined prevalence of *Aspergillus* coinfection among patients with pulmonary tuberculosis was 15.4% [4]. DM is widely recognized as a risk factor for invasive pulmonary aspergillosis. Diabetic patients have an immune system with a lower ability to respond to and deal with aspergillosis, and prolonged hyperglycemia results in unfavorable outcomes in infected patients [5]. *Aspergillus lentulus* is an emerging fungal pathogen in China and invasive aspergillosis due to *A. lentulus* is associated with high mortality [6]. Herein, we report a case of pulmonary coinfection with *M. tuberculosis* and *A. lentulus* in a diabetic patient diagnosed by metagenome next-generation sequencing.

## Case presentation

A 79-year-old man was admitted to the cardiology department of our hospital, complaining of a 7-day history of fever, with a temperature up to 39.5 ℃. He denied cough, phlegm, nasal obstruction, pharyngalgia, chest pain, dizziness, or headache. Five days before admission, he was diagnosed with pulmonary infection by chest X-ray and given antibiotic therapy at a local hospital, but his symptoms did not relieve. The patient was diagnosed with diabetes mellitus two months ago and drank 2000–3000 ml of water per day. About 20 days ago, he began to present chest tightness and shortness of breath after physical activity. In addition, the patient had a history of high blood pressure for more than 20 years.

**Fig. 1:**
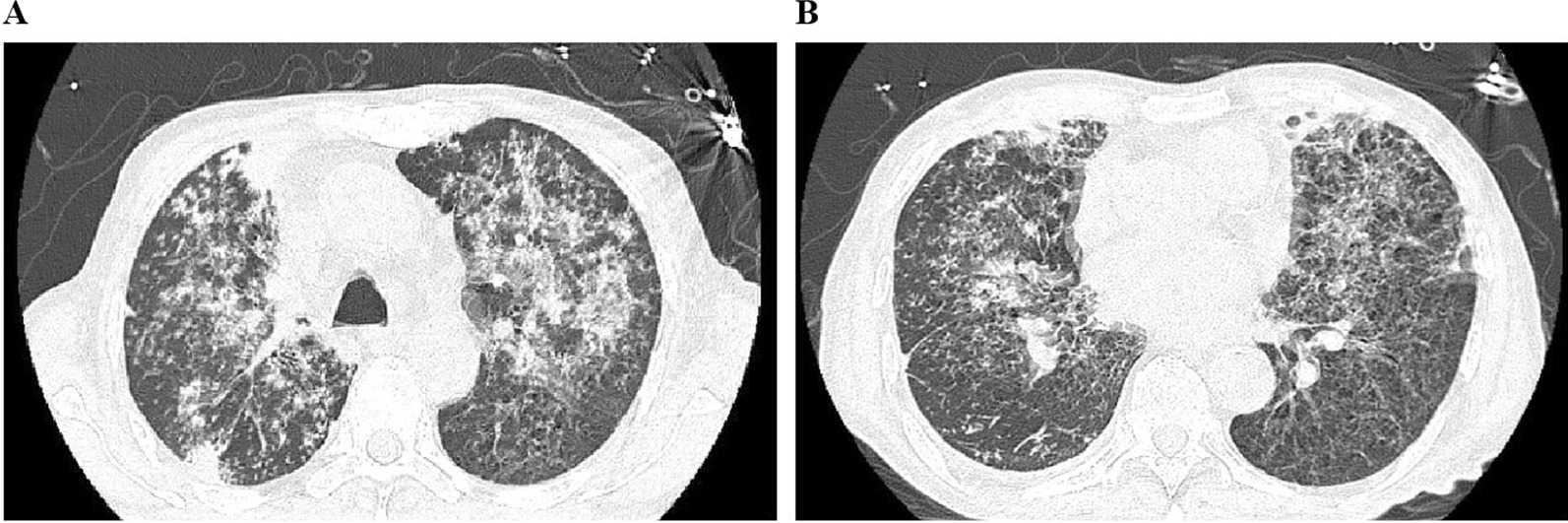
Images of chest CT on admission. **A**, **B** Chest CT scan showed multiple nodules and patchy infiltration in both lungs

On admission (day 0), physical examination showed conscious state, temperature of 36.5 ℃, heart rate of 78/min, respiratory rate of 20/min, blood pressure of 120/62 mmHg, and crackles on pulmonary auscultation. The patient reported a weight loss of 10 kg in the last six months. Laboratory investigations showed white blood cell of 7.77 × 10^9^/L (with 90.1% neutrophil, 2.5% monocytes, and 7.2% lymphocytes), procalcitonin of 3.24 ng/ml, erythrocyte sedimentation rate of 52 mm/h, hemoglobin A1c of 9.5%, and fasting blood sugar of 8.26 mmol/L. A chest computed tomography (CT) scan showed multiple nodules and patchy infiltration in both lungs (Fig. 1A, B), which did not exclude the possibility of tuberculosis. The preliminary diagnosis was pulmonary infection, and empiric antibiotic treatment with intravenous piperacillin/tazobactam (4.5 g, q12h) and moxifloxacin (2 g, q12h) was given. Intravenous insulin (4 U, qd) and oral miglitol (50 mg, q12h) were used to control blood glucose level.

After admission, the patient developed continuous fever and daily temperature peak exceeded 38.5 ℃. On day 1, he developed respiratory failure and received oxygen treatment. Arterial blood gas analysis showed PH 7.50, PaO_2_ of 57.7 mmHg, PaCO_2_ of 31.9 mmHg, and SaO_2_ of 90.2%. Serological tests for influenza A IgM, influenza B IgM, parainfluenza virus IgM, adenovirus IgM, respiratory syncytial virus IgM, *Mycoplasma pneumoniae* IgM, and *Chlamydia pneumoniae* IgM proved negative. Tuberculosis immune spot test was positive. On day 2, the patient presented polyuria and polydipsia, and his symptom of tachypnea was not relieved during oxygen treatment. Then he was transferred to the intensive care unit (ICU) and given mechanical ventilation. On day 3, the patient had a PASS score of − 1, and received analgesia and sedation with propofol and alfentanil. Bronchoscopy found bronchial mucosal inflammation and production of purulent sputum, and bronchoalveolar lavage fluid (BALF) was obtained for further detection.

On day 5, the patient underwent a decreased oxygenation index (155) compared with before. No abnormalities were found in his blood culture. Serum galactomannan (1.74 ng/ml) and 1,3-β-d-glucan (424.50 ng/L) were positive. Acid-fast staining and culture of BALF were negative. *M. tuberculosis* DNA detection from BALF was positive. The BALF sample was also sent for metagenomic next-generation sequencing (Genskey, Beijing, China). A total of 86,398,947 raw reads were generated and 60,161,047 high quality reads were obtained after removing low quality reads. Among the high quality reads, 47,232,566 were aligned with the human reference genome (HG38) and 12,928,481 were used for downstream analysis. The mNGS of BALF reported *M. tuberculosis* complex (7,453,374 reads), *A. lentulus* (37,868 reads), and several oral colonizing microorganisms. Metagenome data is now available at NCBI under the Sequence Read Archive (SRA) database with accession no. PRJNA917778. Based on these findings, the patient was diagnosed as coinfection of *M. tuberculosis* and *A. lentulus.* Initial antibiotic treatment was changed to oral isoniazid (0.3 g, qd), rifapentine (0.45 g, qd) for anti-tuberculosis, and intravenous caspofungin (50 mg, qd) for anti-fungal.

On day 6, the patient's condition aggravated again. He developed multiple organ dysfunction and fell into a light coma state. His family decided to abandon treatment because of financial problems and grave prognosis. Artificial respiration was stopped subsequently and the patient expired 1.5 h later.

## Discussion and conclusion

TB and DM are both significant public health problems in China, and they have mutual risk factors. A higher prevalence of TB among DM patients and vice versa were found in many previous studies worldwide [7]. TB and DM patients are usually immunocompromised and susceptible to fungal infections. Among patients with *M. tuberculosis* and *Aspergillus* coinfection, the most frequency of *Aspergillus spp.* was *A. fumigatus* with a prevalence of 57.6% [4]. Here, we report a rare case of pulmonary infection caused by *M. tuberculosis* and *A. lentulus* in a diabetic patient, and believe that *A. lentulus* infection was secondary to TB and DM.

Pulmonary TB and aspergillosis have similar clinical symptoms including fever, chest tightness, shortness of breath, cough, chest pain, sputum with blood streaks, weight loss, and night sweats [8]. Therefore, they might be misdiagnosed and mistreated in clinical practice, and a definitive diagnosis relies on etiological detection results. However, traditional methods often have limitations for the detection of rare pathogens or mixed infections.

In recent years, the application of mNGS shed light on detecting pathogens much faster and more efficiently. Miao et al. demonstrated that the mNGS method had higher sensitivity and specificity than microbial culture, especially for the detection of *M. tuberculosis*, fungi, anaerobic bacteria, and viruses [9]. As an unbiased and rapid diagnostic method, mNGS can sequence thousands to billions of DNA fragments and provide information on a broad spectrum of organisms simultaneously.

mNGS has a great advantage for the diagnosis of mixed pulmonary infection. Mixed pulmonary infection is defined when coinfection with two or more pathogens is identified. A study showed that the sensitivity of mNGS in mixed pulmonary infection diagnosis was seven times higher than that of conventional tests (97.2% vs 13.9%) [10]. Another study showed that the rate of confirmed mixed pulmonary infection detected by mNGS was four times higher than that detected by conventional methods [11]. Yan et al. reported a case on mixed pulmonary infection of *Nocardia nova*, *M. tuberculosis*, and *A. fumigatus* based on mNGS. Although sputum and BALF were sent several times for smear and culture, only *A. fumigatus* was detected by culture [8]. Qin et al. reported a case of mixed pulmonary infection with *Corynebacterium striatum*, *Pseudomonas aeruginosa*, *Streptococcus pneumoniae*, and *Cryptococcus neoformans* through mNGS*,* while microbial culture and immunological tests were all negative [12]. Mixed pulmonary infection is very common in clinical experience, especially in immunocompromised patients, so early accurate diagnosis and appropriate treatment may reduce the mortality.

Invasive aspergillosis remains a major invasive fungal infection with serious clinical consequences among immunocompromised patients, and *A. fumigatus* is the most common cause [13]. As a sibling species of *A. fumigatus*, *A. lentulus* was first described in 2004, which caused fatal infections in four hematopoietic stem cell transplant patients [14, 15]. This species is usually difficult to distinguish from *A. fumigatus* based on phenotypic typing, and can be misidentified as *A. fumigatus* [16]. *A. lentulus* is genetically distinct from *A. fumigatus* and highly drug resistant. Molecular identification and matrix-assisted laser desorption ionization-time of flight mass spectrometry (MALDI-TOF MS) can be used to distinguish them [17].

*A. lentulus* tends to cause infections associated with high mortality (> 60%) and poor clinical outcomes [18]. In China*,* the first *A. lentulus* strain was isolated from a patient with acute pulmonary infection in 2011, who died due to aggressive lung fungal infection after active treatment [19]. In 2020, Yu et al*.* reported six patients with proven or probable *A. lentulus* infection from China. Four of the patients had poor prognosis. All the six isolates were in vitro resistant to multiple anti-fungal drugs, including amphotericin B, itraconazole, voriconazole, and posaconazole. Immunocompromise, critical illness, and prior anti-fungal were common risks for invasive *A. lentulus* infection [6]. Although *A. lentulus* infection is not commonly found in China, we still need to pay attention to its pathogenicity in clinical practice.

Collectively, we report the first case of pulmonary coinfection caused by *M. tuberculosis* and *A. lentulus* in China, to the best of our knowledge. Clinicians and laboratories should be alert to the emerging *A. lentulus* infection in China due to its drug-resistance and high mortality. In comparison with conventional methods, mNGS has a great advantage for the diagnosis of mixed pulmonary infection.

## Data Availability

All data generated or analysed during this study are included in this published article.

## Abbreviations

mNGS: Metagenome next-generation sequencing
DM: Diabetes mellitus
TB: Tuberculosis
CT: Computed tomography
ICU: Intensive care unit
BALF: Bronchoalveolar lavage fluid
MALDI-TOF MS: Matrix-assisted laser desorption ionization-time of flight mass spectrometry
